# RNA-Seq and Comparative Transcriptomic Analyses of Asian Soybean Rust Resistant and Susceptible Soybean Genotypes Provide Insights into Identifying Disease Resistance Genes

**DOI:** 10.3390/ijms241713450

**Published:** 2023-08-30

**Authors:** Qingnan Hao, Hongli Yang, Shuilian Chen, Yanhui Qu, Chanjuan Zhang, Limiao Chen, Dong Cao, Songli Yuan, Wei Guo, Zhonglu Yang, Yi Huang, Zhihui Shan, Haifeng Chen, Xinan Zhou

**Affiliations:** 1 Institute of Oil Crops Research, Chinese Academy of Agriculture Sciences, Wuhan 430062, China; haoqingnan@caas.cn (Q.H.); yanghongli@caas.cn (H.Y.); chenshuilian@caas.cn (S.C.); q1098789459@163.com (Y.Q.); zhangchanjuan@caas.cn (C.Z.); chenlimiao@caas.cn (L.C.); caodong@caas.cn (D.C.); songliyuan@caas.cn (S.Y.); guowei03@caas.cn (W.G.); yangzhonglu@caas.cn (Z.Y.); huangyi@caas.cn (Y.H.); zhouxinan@caas.cn (X.Z.); 2 Key Laboratory for Biological Sciences of Oil Crops, Chinese Academy of Agricultural Sciences, Ministry of Agriculture and Rural Affairs, Wuhan 430062, China; 3 The Graduate School of Chinese Academy of Agricultural Sciences, Beijing 100081, China

**Keywords:** soybean, Asian soybean rust, RNA-seq, WGCNA

## Abstract

Asian soybean rust (ASR), caused by *Phakopsora pachyrhizi*, is one of the most destructive foliar diseases that affect soybeans. Developing resistant cultivars is the most cost-effective, environmentally friendly, and easy strategy for controlling the disease. However, the current understanding of the mechanisms underlying soybean resistance to *P. pachyrhizi* remains limited, which poses a significant challenge in devising effective control strategies. In this study, comparative transcriptomic profiling using one resistant genotype and one susceptible genotype was performed under infected and control conditions to understand the regulatory network operating between soybean and *P. pachyrhizi*. RNA-Seq analysis identified a total of 6540 differentially expressed genes (DEGs), which were shared by all four genotypes. The DEGs are involved in defense responses, stress responses, stimulus responses, flavonoid metabolism, and biosynthesis after infection with *P. pachyrhizi*. A total of 25,377 genes were divided into 33 modules using weighted gene co-expression network analysis (WGCNA). Two modules were significantly associated with pathogen defense. The DEGs were mainly enriched in RNA processing, plant-type hypersensitive response, negative regulation of cell growth, and a programmed cell death process. In conclusion, these results will provide an important resource for mining resistant genes to *P. pachyrhizi* infection and valuable resources to potentially pyramid quantitative resistance loci for improving soybean germplasm.

## 1. Introduction

The challenge of maintaining and increasing the global food supply is a significant concern for human civilization in the 21st century. Maximizing agricultural crop yields has always been a formidable task due to the presence of pathogens. Soybean (*Glycine max*), being one of the most important crops globally, is extensively cultivated and serves as the primary legume crop that supplies 70% of the world’s protein meal as well as food oil and is a sustainable fuel source (www.soystats.com, accessed on 1 May 2023). The occurrence of Asian soybean rust (ASR), caused by the obligate biotrophic fungus *Phakopsora pachyrhizi*, leads to significant yield losses of up to 90% in all soybean-producing countries [1]. ASR exhibits a wide host range and has the ability to infect over 150 plants belonging to 53 different genera, and soybean is the best host [2]. The primary pathogenic forms of rust bacteria are summer spores. The dissemination of soybean rust is facilitated by wind and characterized by strong fulmination, rendering control efforts challenging. This disease manifests severely in regions with high temperatures and humidity and has emerged as a primary affliction of soybean crops in tropical and subtropical locales [3]. In South America, soybean rust is estimated to affect approximately 80% of the total cultivated area of soybean crops, with its prevalence being ubiquitous in Latin American regions, where an estimated 210 million metric tons of soybeans are expected to be produced by 2022/23, as per incomplete statistics (https://apps.fas.usda.gov/psdonline/app/index.html, accessed on 14 March 2023), and on average, represent a gross production value of USD 115 billion per season (https://www.ers.usda.gov/data products/season-average-price-forecasts.aspx, accessed on 14 March 2023). Chemical control is the only method by which to control the disease due to the limited resistance available in soybean germplasm. In Brazil, the estimated cost for controlling *P. pachyrhizi* is approximately USD 2.8 billion per season (http://news.agropages.com/News/NewsDetail-33194.htm, accessed on 14 March 2023) [4]. To address this issue, the most cost-effective and efficient approach is to select and breed superior varieties with a high yield and stable and persistent resistance, which can effectively control ASR in soybeans and other crops [5].

Over 30,000 soybean resources have been identified for their resistance to rust [6,7,8,9,10,11]. However, only eight genetic loci associated with resistance have been identified thus far, and a limited number of 20 resistance resources with distinct genetic characteristics have been discovered. Furthermore, it has been observed that existing ASR isolates have the ability to overcome the resistance provided by six loci [12,13]. Eight major resistance loci that confer resistance to *Phakopsora pachyrhizi* are *Rpp1* [14], *Rpp2* [15], *Rpp3* [16], *Rpp4* [17], *Rpp5* [18], *Rpp6* [19], *Rpp7* [20] and *Rpp6907* from the resistant material SX6907 in our lab [21]. A limited number of ASR-resistant genes have been successfully cloned. Although certain cultivars can acquire rust resistance through backcross transfer, conventional breeding techniques heavily depend on the accessibility of disease-resistant resources and are hindered by pathogen evolution, posing significant challenges to substantial advancements. Given the limited genetic diversity and absence of resistant germplasm, addressing this lethal disease has emerged as a formidable predicament for scientific researchers worldwide.

Plants demonstrate their immune response through a series of signaling pathways that encompass recognition, signal transduction, and downstream defense reactions, such as the production of antibacterial compounds [22]. RNA-seq technologies are instrumental in investigating the intricate biological processes at a transcriptional level [23], as they have significantly enhanced gene discovery efficiency while reducing time, labor, and cost. Recently, RNA-Seq was employed to elucidate the transcriptional resistance mechanisms to plant pathogens (e.g., [24,25,26]; however, up to now, the response of soybean to *P. pachyrhizi* still has only a few reports. Panthee et al. [27] discovered notable variations in gene expression during different growth stages of susceptible soybean crops in response to *P. pachyrhizi* by utilizing whole-genome Affymetrix microarrays. Tremblay et al. [28] employed mRNA-Seq to scrutinize the pathogen’s transcript abundance at specific time points, namely 15 s, 7 h, 48 h, and 10 d after the inoculation of susceptible soybean leaves with *P. pachyrhizi*, with the objective of obtaining fresh insights into the transcript abundance in soybean and the pathogen, including the uredinial stage, during the infection. Hossain et al. [1] have provided an elucidation of the variation in *Rpp3* gene-mediated resistance to *P. pachyrhizi* by focusing on the major genes of a phenylpropanoid pathway through RNA-seq. Therefore, it is crucial to concentrate on the primary infection stages for the examination of the defense responses. A comparative analysis of transcriptional profiling of soybean varieties with contrasting disease resistance will offer a comprehensive understanding of the response of resistant varieties to *P. pachyrhizi* infection and enhance the breeding of resistant varieties for more effective control of *P. pachyrhizi*.

In a prior investigation, numerous Chinese soybean germplasm resources were assessed to ascertain their level of resistance to *P. pachyrhizi*. Of these resources, only SX6907 exhibited immunity to local mixed *P. pachyrhizi* isolates [7]. The current study endeavors to enhance comprehension of the molecular mechanism underlying the resistance conferred by SX6907 against *P. pachyrhizi*. To achieve this objective, we are conducting a comparative analysis of the transcriptome of resistant and susceptible *P. pachyrhizi* accessions to elucidate the molecular foundation of resistance and susceptibility. The primary objective of this study was to identify genes that are potentially implicated in conferring resistance against *P. pachyrhizi*. The findings of this investigation are expected to furnish the molecular underpinnings for the development of resistant cultivars and the formulation of disease management tactics while also augmenting the current knowledge on mechanisms of disease resistance.

## 2. Results

### 2.1. The Phenotypic Response of Soybean Accessions to P. pachyrhizi

**Figure 1:**
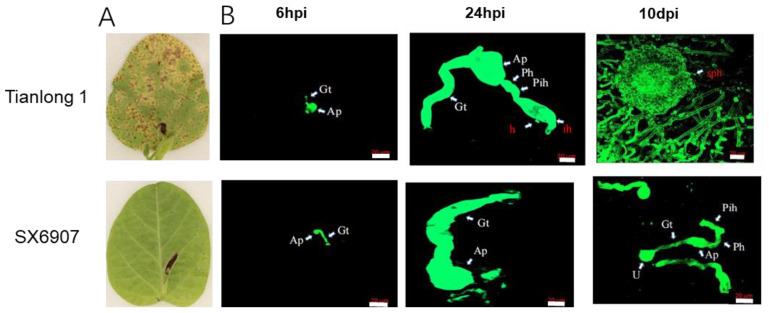
Asian soybean rust resistant phenotypes’ microscopic observation of SS4 growth. (**A**) Asian soybean rust resistant phenotypes of SX6907 and tianlong1 at seedling stages. (**B**) Microscopic observation of pathogen development in SX6907 and tianlong1 at different time points after SS4 infection. Urediospores (u), germ tube (Gt), appressorium (Ap), primary invasive hypha (pih), penetration hypha (ph), invasive hyphae (ih), sporogenous hyphae (sph). Scale bar is 20 μm.

Leaves of SX6907 and tianlong1 seedlings were inoculated with *P. pachyrhizi* isolate SS4. At 10 dpi (days post-inoculation) of *P. pachyrhizi*, it was observed that SX6907 displayed high levels of resistance and no visible lesions, while numerous lesions spread on those of tianlong1 (Figure 1A). According to the results of the seedling incubation assay, SX6907 showed a strong immune response against isolate SS4, whereas tianlong1 was completely susceptible.

An examination of *P. pachyrhizi* growth on SX6907, a resistant cultivar, and tianlong1, a susceptible cultivar, demonstrated a nearly identical developmental process at 24 h post-inoculation. The formation of *P. pachyrhizi* structures within leaf tissues, including germ tube (Gt), appressorium (Ap), primary invasive hypha (pih), penetration hypha (ph), invasive hyphae (ih), and sporogenous hyphae (sph), occurred at this time point in both SX6907 and tianlong1. However, significant differences in *P. pachyrhizi* development were observed between SX6907 and tianlong1 from 3 to 10 days post-inoculation. At 10 days post-inoculation, SX6907 exhibited substantially larger fungal colonies and a greater abundance of pathogen-feeding structures (Figure 1B). In contrast, the fungal development in SX6907 was very limited.

### 2.2. Analysis of RNA-Seq Data

In order to comprehend the molecular mechanisms of resistance, we collected three replicates of *P. pachyrhizi*-treated and controlled Pp-R and Pp-S samples at 6 hpi, 24 hpi, and 10 d to capture the transcriptional changes during the biotrophic and transition phases. After adaptor removal and quality filtering, the results of the RNA-seq analysis generated a total of 147.2934 Gbp clean bases of data (983.9058 M reads) from the eight transcriptome samples, including resistant control (R-0 hpi), resistant treatment 6 h (R-6 hpi), resistant treatment 24 h (R-24 hpi), resistant treatment 10 d (R-10 dpi), susceptible control (S-0 hpi), susceptible treatment 6 h (S-6 hpi), susceptible treatment 24 h (S-24 hpi), and susceptible treatment 10 d (S-10 dpi). A rigorous standard was employed in the data cleaning process, whereby all sequences with a Phred score of 20 were eliminated, resulting in a set of clean reads ranging from 37 to 45 million across all samples (Table 1). The clean reads from all 24 libraries were subsequently aligned to the soybean genome Wm82.a4.v1, with an overall mapping coverage of 70–93% observed across all samples. The percentage of uniquely mapped reads ranged from 69% to 90% for all samples. Subsequent analysis was conducted solely on the uniquely mapped reads, with the comprehensive sequencing and mapping statistics presented in Table 1. The transcriptome data of all samples qualified for further analysis to identify the differentially expressed genes (DEGs).

Genes with reads per kilobase million (RPKM) values exceeding 1 in three replicates for each group were considered to be expressed. The treated and control groups of both genotypes at three time points collectively expressed more than 30,000 genes, with a varying number of specifically expressed genes in each group (Supplementary Table S1). In total, 258,567 expressed genes were identified across all eight groups, with 132,190 genes expressed in the Pp-R samples and 126,377 genes expressed in the Pp-S samples.

### 2.3. Validation of Selected Differentially Expressed Genes (DEGs) Using qRT-PCR

**Figure 2:**
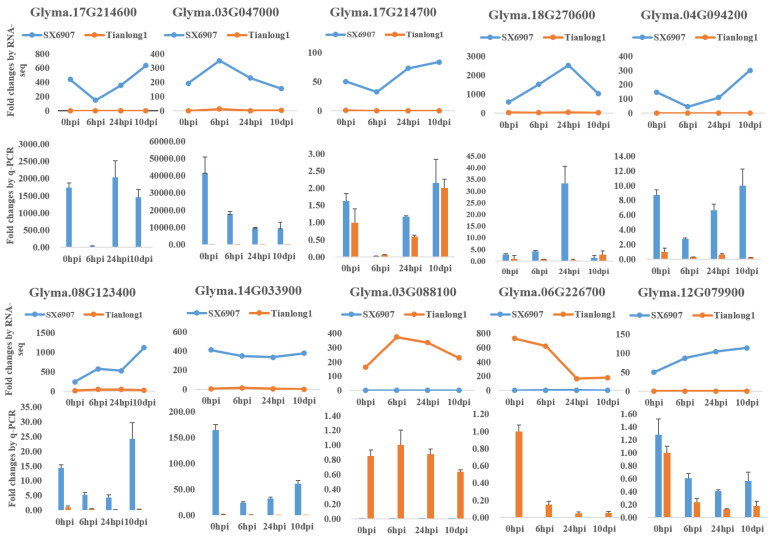
The relative gene expression of 10 randomly selected genes examined by quantitative real-time PCR and RNA-seq.

In order to verify the differential expression analysis based on RNA-seq data, the transcriptional levels of the selected DEGs were evaluated via qRT-PCR analysis. These DEGs were deemed to be potentially involved in defense against *P. pachyrhizi* and exhibited distinct expression profiles across both the resistant and susceptible samples treated with *P. pachyrhizi* and the control samples. A total of 10 DEGs were selected for qRT-PCR analysis (Figure 2). The results of the qRT-PCR analysis were in good agreement with the RNA-seq data (Figure 2), thereby validating the reliability of the gene expression levels obtained through the DGE analysis.

### 2.4. Comparative Analysis of Tianlong1 and SX6907 Transcriptomes

**Figure 3:**
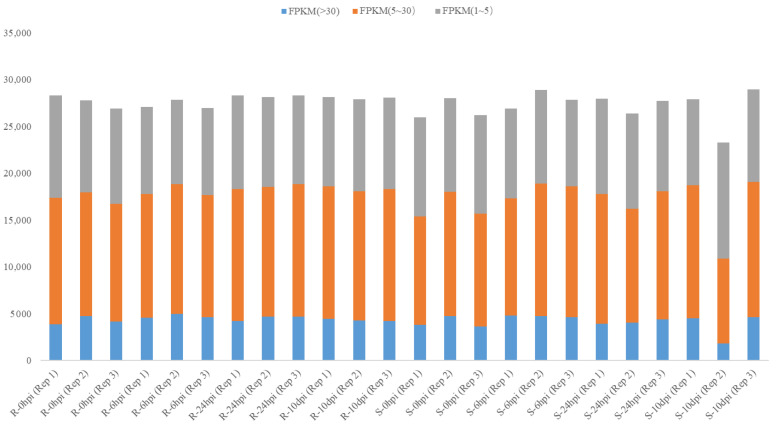
Whole genomic gene expression for SX6907 and tianlong1 inoculation with *P. pachyrhizi* at 0 h, 6 h, 24 h, and 10 d. The y-axis represents the gene number. The blue box means fragments per kilo bases per million reads (FPKM) > 30, the orange box means FPKM 5–30, and the gray box means FPKM 1–5.

This present study compared the whole genomic gene expression of tianlong1 and SX6907 at four distinct time points. The analysis revealed that the average expressional intensity (measured in fragments per kilo bases per million reads (FPKM) > 1) of whole genomic genes was highest at 24 hpi for SX6907 and at 6 hpi for tianlong1, in comparison to the other time points (Figure 3).

**Figure 4:**
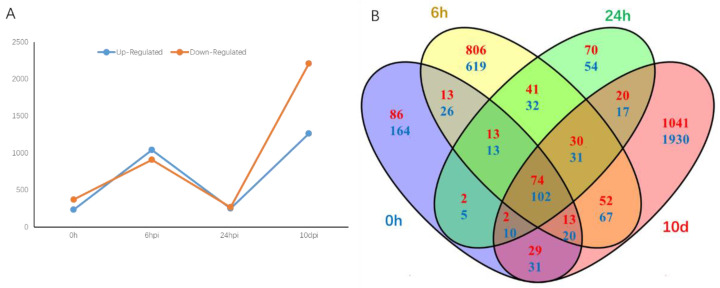
Number of differentially expressed genes in soybeans infected by Asian soybean rust. (**A**) The number of differently expressed genes in SX6907 compared with tianlong1 at four time points (0 h, 6 h, 24 h, and 10 d). The y-axis represents the DEGs number. Number of DEGs was counted with the criteria *p* < 0.05 and log2 (fold change) > 1. (**B**) Venn diagram comparison of differentially expressed genes (assigned by *p* < 0.05 and log2 (fold change) > 1) at four infection stages (0 h, 6 h, 24 h, and 10 dpi) in SX6907 compared with tianlong1. Red highlighted numbers represent the amount of upregulated DEGs, and blue highlighted numbers represent the amount of downregulated DEGs.

The quantification of the differentially expressed genes (DEGs) between SX6907 and tianlong1, used as the control, was conducted at each time point. The expression patterns of the DEGs were classified into upregulated and downregulated groups. In the 0 h libraries, the count of upregulated genes was lower compared to that of the downregulated genes. However, after 6 h of inoculation, the count of the upregulated genes exceeded that of the downregulated genes. The highest count of DEGs was observed 10 days post-inoculation (Figure 4A).

The present study utilized Venn diagrams to compare the differential expression of genes between SX6907 and tianlong1 (used as control) after inoculation of rust fungi. The results indicated that at 10 dpi, 1041 upregulated and 1930 downregulated DEGs were specifically detected, while at 24 hpi, only 70 upregulated and 54 downregulated DEGs were specifically detected. Similarly, at 6 hpi, 806 upregulated and 619 downregulated DEGs were specifically detected. Notably, the least number of differentially expressed genes was detected at 24 hpi. Overall, a total of 74 upregulated and 102 downregulated DEGs were detected at all four time points (Figure 4B). The results indicate that some DEGs are dependently regulated on the infection time, and some are independently regulated.

**Figure 5:**
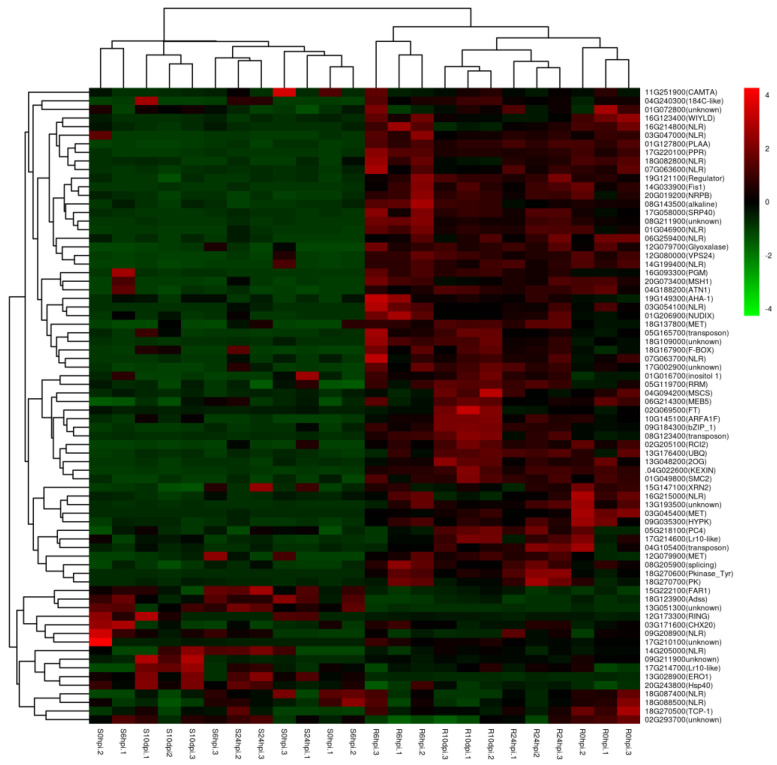
Heat map showing hierarchical cluster analysis of 74 expressed genes across all samples. Gradient scale represents expression levels, with red showing the highest expression to greenwith the lowest expression.

A heat map was generated through hierarchical cluster analysis of the expressed genes across all samples, which unveiled distinct expression patterns of certain disease-resistant genes between the Pp-R and Pp-S accessions. Among the 74 upregulated DEGs across all samples, 56 clustered together to reveal higher expression levels in all four R samples compared to the very low expression in all S samples (Figure 5). Among the rest of the clusters, 18 genes were seen as highly induced in susceptible accessions upon *P. pachyrhizi* treatment.

In the *P. pachyrhizi*-treated samples, 104 genes were found to be the strongest, upregulated in Pp-R, and comparatively less induced in Pp-S at all 6 hpi, 24 hpi, and 10 dpi. Some genes have an annotation correlated with disease resistance being listed (Table 2). Fifteen were LRR and NB-ARC domain-containing disease-resistant proteins; two genes were the rust resistance kinase Lr10 isoform. A total of 133 genes were found to be highly downregulated in Pp-R and comparatively less induced in Pp-S at all 6 hpi, 24 hpi, and 10 dpi. A total of 17 were LRR and NB−ARC domain-containing disease-resistant proteins; 7 were the LRR receptor protein kinase; 2 were rust resistance kinase lr10-related protein; 2 were programmed cell death protein; and 2 were the TMV resistance protein N isoform (Table 2).

### 2.5. Gene Ontology (GO)-Based Analysis of the DEGs

**Figure 6:**
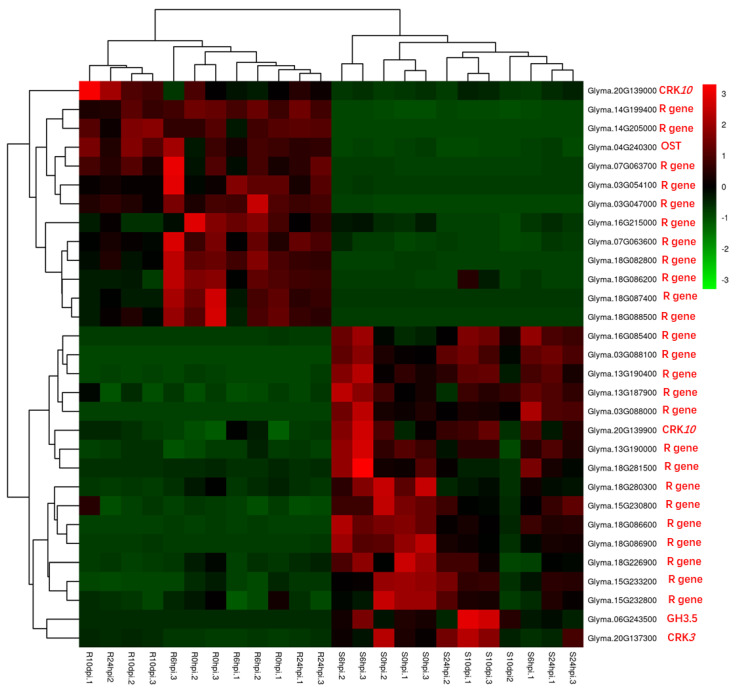
Heat map showing expression profiles of core defense genes that could be putatively involved in resistance development against *P. pachyrhizi* in Pp−R. Gradient scale shows Z−scores of DEGs where red represents the most induced expression and green depicts the highest repression. Abbreviations: CRK−cysteine-rich receptor-like protein kinase; GH−indole-3-acetic acid−amido synthetase; OST−organic solute transporter; R gene−NB-ARC domain-containing disease-resistant gene.

Gene set enrichment analysis was employed to identify the functional gene categories (GO terms) and determine the significant biological processes in SX6907 compared to tianlong1 under *P. pachyrhizi* infection. The results revealed that defense response was the functional category that was significantly over-represented at all four time points (Table 3, Supplementary Table S2). Additionally, a heat map was generated to display the 30 expression profiles of the core defense genes at all four time points (Figure 6). Notably, 11 NB−ARC domain-containing R genes were expressed at high levels solely in Pp−R in both the control and treated samples. Fourteen R genes containing NB-ARC domains were expressed at a low level, exclusively in Pp-R, in both the control and treated samples. Three receptor-like protein kinases rich in cysteine were found to have inducible expression. The categories of response to stress and response to stimulus were over-represented at 0 hpi, 6 hpi, and 24 hpi. In contrast, the categories of secondary metabolic process, protein phosphorylation, oxidation-reduction process, and response to biotic stimulus were over-represented among the transcripts at 10 dpi. Significant differential induction of these core defense components in Pp−R compared to Pp−S makes them the potential candidates responsible for resistance against *P. pachyrhizi* in Pp−R accession.

### 2.6. KEGG Pathway Analysis of the DEGs

**Figure 7:**
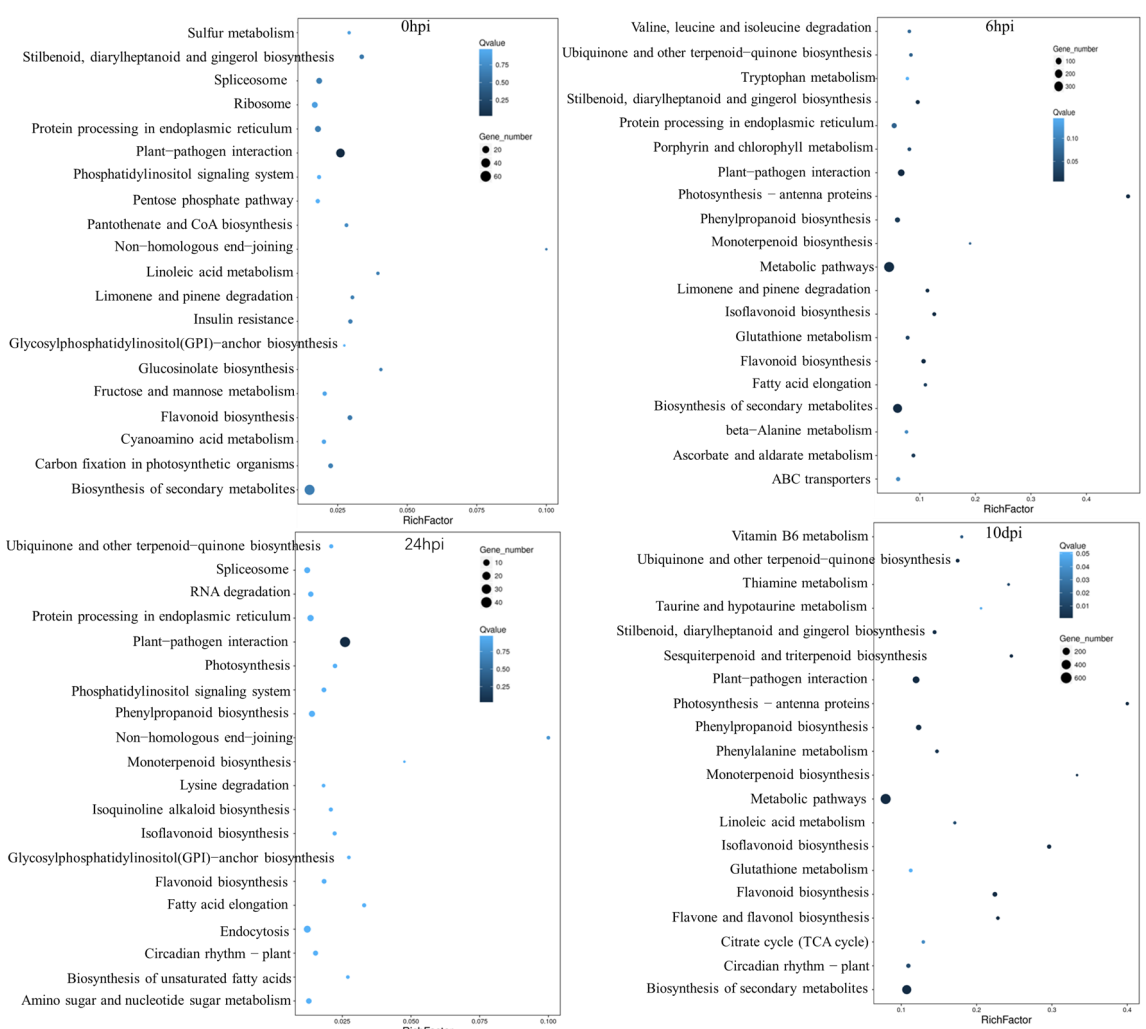
The KEGG pathway enrichment analysis of DEGs at four time points to *P. pachyrhizi* infection.

The Kyoto Encyclopedia for Genes and Genomes (KEGG) enrichment analysis was conducted to identify the main metabolic pathways associated with plant defense in the SX6907 infected by *P. pachyrhizi*. The DEGs were mapped to canonical pathways in the KEGG database. Based on the number of enriched DEGs, we identified the top 20 pathways (Figure 7). A total of 379 DEGs were mapped with the metabolic pathways related to plant defense at 6 hpi, which is a key time point for the most DEGs detected at this point (Table 4). The plant–pathogen interaction pathway and flavonoid biosynthesis were significantly enriched at all four time points. A total of 207 genes were mapped in the plant–pathogen interaction pathway. A total of 61 genes were mapped in the flavonoid biosynthesis pathway. In addition, the results indicated a large number of DEGs were enriched in the plant hormone signal transduction pathways. NPR1, TGA and PR-1 in the salicylic acid pathway were found to be upregulated in Pp-R and downregulated in Pp−S at 6 hpi. JAZ was found to be upregulated in Pp−R and downregulated in Pp-S at three time points. JAR1 and BAK1 were found to be downregulated in Pp−R and upregulated in Pp−S at three time points (Supplementary Figure S1).

### 2.7. Identification of Transcription Factors

**Figure 8:**
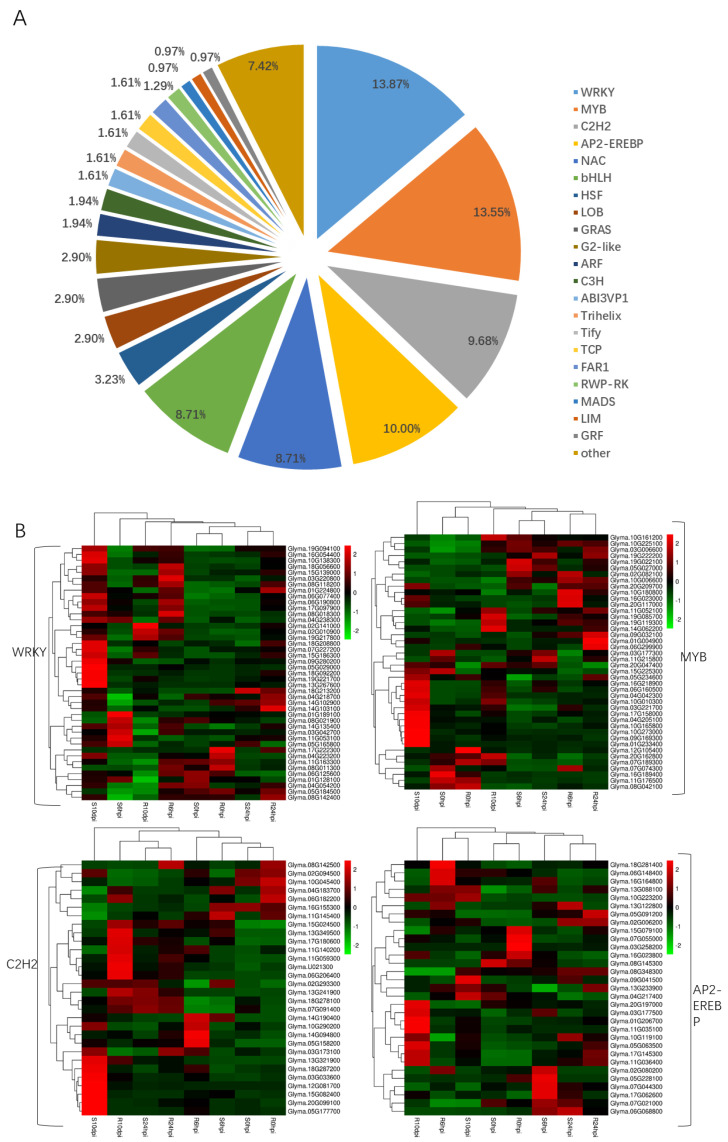
Analysis of differentially expressed transcription factors in four time points. (**A**). The ratios of differentially expressed transcription factor families. (**B**). Expression levels are shown for the WRKY, MYB, C2H2, and AP2−EREBP transcription factors in resistant material and susceptible material. FPKM values are represented by color gradient. Too high = red brick.

Transcription factors (TFs) play a crucial role in responding to biotic stresses, such as insect attacks and pathogen infections [29]. The present study has identified 311 DEGs that are annotated as transcription factors, belonging to 37 distinct transcription factor families, including the WRKY, MYB, C2H2, AP2−EREBP, NAC, bHLH, HSF, LOB, GRAS, G2-like, ARF, C3H, ABI3VP1, Trihelix, Tify, TCP, FAR1, RWP-RK, MADS, LIM, GRF, TAZ, SBP, PLATZ, mTERF, HB, DBP, CAMTA, BES1, ULT, Sigma70-like, LFY, E2F−DP, CSD, CPP, and bZIP transcription factors (Supplementary Table S3). The most prevalent transcription factors were those belonging to the WRKY family, which were encoded by 43 DEGs, accounting for 14% of the total 311 differentially expressed transcription factors. This was followed by the MYB transcription factors (42 DEGs, 14%), AP2-EREBP transcription factors (31 DEGs, 10%), and C2H2 transcription factors (30 DEGs, 10%). The distribution of these transcription factor families is illustrated in Figure 8A. The WRKY, MYB, AP2-EREBP, and C2H2 transcription factors may have a significant role in responding to pathogen attacks. Subsequently, we conducted a cluster analysis on the shared differential transcription factors in both materials (Figure 8B). Upon further analysis, it was observed that Glyma.11G251900 (CAMTA), Glyma.15G154500 (mTERF), and Glyma.15G222100 (FAR1) exhibited differential expression across all four time points. Glyma.08G360200 (NAC), Glyma.15G078300 (NAC), and Glyma.16G182400 (RWP−RK) displayed differential expression at 6 hpi and 24 hpi, while Glyma.05G191300 (NAC) exhibited a common differential expression at 6 hpi, 24 hpi, and 10 dpi. Glyma.06G250300 (ULT) and Glyma.13G112000 (Tify) were differentially expressed at 24 hpi and 10 dpi. Furthermore, 13 transcription factors were exclusively differentially expressed at 0 hpi. At 6 hours post-inoculation, 87 transcription factors exhibited differential expression, while at 24 hours post-inoculation, 6 transcription factors showed differential expression, and at 10 days post-inoculation, 172 transcription factors displayed differential expression. The analysis revealed that 16 transcription factors (6 WRKY, 4 HSF, 2 C2H2, 3 MYB, and 1 C2C2−Dof) were upregulated at 6 hours post-inoculation but downregulated at 10 days post-inoculation. Additionally, one AP2−EREBP transcription factor (Glyma.11G036400) was downregulated at 6 hours post-inoculation but upregulated at 10 days post-inoculation. One Tify transcription factor (Glyma.13G112000) was upregulated in 24 hpi but downregulated at 10 d post−inoculation. The transcription factors with inconsistent expression trends may play an important role in the resistance of *P. pachyrhizi*.

### 2.8. Co-Expression Network Analysis of Soybean Resistance to P. pachyrhizi

**Figure 9:**
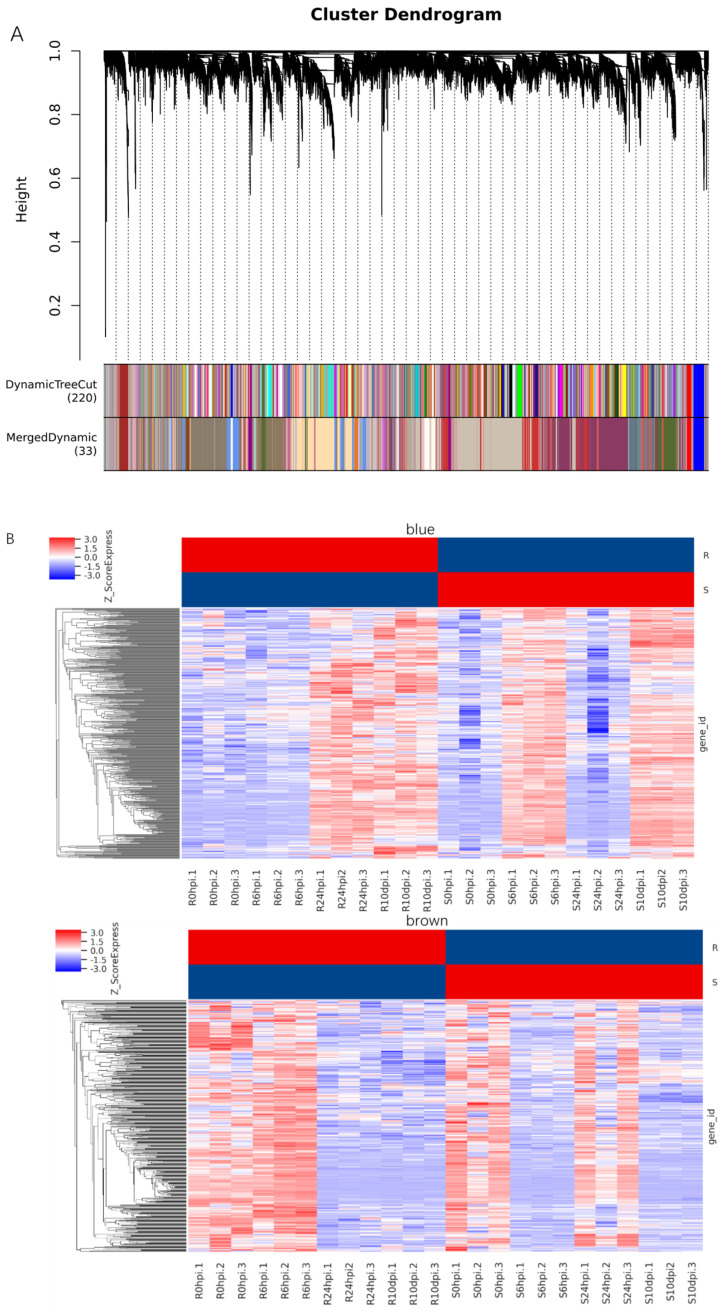
Weighted gene co-expression network analysis (WGCNA) of differentially expressed genes. (**A**) Dendrogram of all differentially expressed genes clustered based on a dissimilarity measure (1−TOM). The upper part of the figure is the gene cluster Tree constructed by dissTOM matrix constructed by weighted correlation coefficients; the lower part of the figure is divided into the distribution of genes in each module, the same color represents the same module; the Dynamic TreeCut color is the module identified by dynamicTreeCut method. (**B**) Hierarchical clustering analysis of all differentially expressed genes (DEGs) in particular interest modules. Blue color represents downregulation of genes. Red indicates upregulation of genes.

In order to enhance the comprehension of the correlation between gene expression and the susceptibility or resistance of leaves to *P. pachyrhizi* and to ascertain the genes that are exclusively linked to resistance to *P. pachyrhizi*, the co−expression networks of 24 RNA-Seq datasets comprising leaf samples at four different time points with three replicates were analyzed using weighted gene co−expression network analysis (WGCNA). This analysis resulted in the identification of 24,386 DEGs. A WGCNA model was established using the chosen power values, resulting in the division of 24,386 genes into 33 modules. The module sizes ranged from 30 to 4436, with the exception of the “Grey” module, which comprised genes that could not be assigned to any module and lacked reference significance (Figure 9A).

The “blue” and “brown” modules were highly associated with an individual infected stage between SX6907 and tianlong1, and the two modules were selected as the particular interest module for further analysis (Figure 9B,  Supplementary Figure S2). A total of 451 genes were clustered in the “blue” module, which were differentially expressed between SX6907 and tianlong1 at 6 hpi and 24 hpi. These were categorized into three functional groups (BP, CC, and MF). The “blue” module in the BP group was mainly enriched in RNA processing (GO:0006396), plant−type hypersensitive response (GO:0009626), host-programmed cell death induced by symbiont (GO:0034050), cell death (GO:0008219), negative regulation of cell growth (GO:0030308), and programmed cell death (GO:0012501). The genes in the MF group were mainly enriched in the structural constituent of the cytoskeleton (GO:0005200) and RNA binding (GO:0003723). The genes in the CC group were significantly enriched in an extrinsic component of the plasma membrane (GO:0019897) and filiform apparatus (GO:0043680) (Supplementary Figure S3A). According to theKEGG pathway analysis, our results demonstrated that these genes were mainly involved in the plant–pathogen interaction (ko04626) and sphingolipid metabolism (ko00600) (Supplementary Figure S3B). A total of 432 genes were clustered in the “brown” module, which was differentially expressed between SX6907 and tianlong1 after infection. The “brown” module in the BP group was mainly enriched in the negative regulation of cell growth (GO:0030308), negative regulation of growth (GO:0045926), regulation of cell growth (GO:0001558), and cell activation involved in immune response (GO:0002263). The genes in the MF group were mainly enriched in 1,3−beta−D−glucan synthase activity (GO:0003843), sterol 24−C−methyltransferase activity (GO:0003838) and ubiquitin modification-dependent histone binding (GO:0061649). The genes in the CC group were significantly enriched in the filiform apparatus (GO:0043680) (Supplementary Figure S3C). The KEGG pathway analysis of our results demonstrated that these genes were mainly involved in the plant–pathogen interaction (ko04626) and sphingolipid metabolism (ko00600) (Supplementary Figure S3D).

## 3. Discussion

### 3.1. Ca^2+^ Signaling Pathway

The influx of Ca^2+^ from the extracellular sources serves as a primary means of signal transduction, with calcium acting as a crucial second messenger ion in eukaryotic organisms [30]. In plants, Ca^2+^ signaling plays a central role in both the pattern-triggered immune (PTI) and effector-triggered immune (ETI) responses, eliciting characteristic cytoplasmic Ca^2+^ elevations in response to the potential pathogens common to both forms of immunity [31]. Our investigation revealed a differential expression of certain genes involved in the regulation of the Ca^2+^ signaling pathway in SX6907 as compared to tianlong1 under *P. pachyrhizi* infection (Supplementary Figure S4 and Table S4), including CNGCs (Glyma.09G168600, etc.), CDPKs (Glyma.02G291300, etc.), and CaM/CML (Glyma.04G194800, etc.). PRR signaling is mediated by the CNGC families [32]. The manifestation of a “defence no death” (DND) phenotype has been observed as a result of loss-of-function mutations in CNGC2 or CNGC4 [33]. This present study has revealed the upregulation of certain CNGC genes at 6 h post-inoculation in the resistant material. Previous research has demonstrated that the disruption of CaM/CML gene expression or loss of CaM/CML function in mutated plants can significantly impair immune responses [34,35,36]. Our findings indicate that a greater number of CaM/CML genes were upregulated in the resistant material in response to *P. pachyrhizi* infection (Supplementary Table S4). Recent research has presented persuasive proof of the participation of CDPKs in the majority of immune signaling occurrences [37]. Our investigation revealed the downregulation of a specific CDPK gene (Glyma.02G291300) in the resistant material following infection with *P. pachyrhizi*. This leads us to hypothesize that the calcium-signaling pathway and its interaction network may be crucial in soybean rust resistance and warrant further examination.

### 3.2. MAPK Signaling Pathway

Plants have not developed adaptive immunity mechanisms similar to those found in animals but have instead evolved a complex system with multiple layers to defend against invading pathogens. The initial response involves the recognition of pathogens by cell-surface pattern-recognition receptors (PRRs), which is known as PAMP−triggered immunity (PTI) [38]. Upon activation of FLS2 and EFR, the MAPK signaling pathway is triggered, leading to the activation of the defense genes responsible for producing antimicrobial compounds. MAPK cascades play a crucial role in regulating various biological processes, including immunity, in plants [39]. The present study utilized RNA−seq analysis to investigate the alterations in gene expression within the MAPK signaling pathway, specifically FLS2, MEKK1, BAK1, EFR, and WRKY, in response to *P. pachyrhizi* infection in soybean. The recognition of flagellin via FLS2 is widely recognized as a crucial component of plant innate immunity [40]. FLS2 (Glyma.03G166300) was found to be upregulated in resistant material, while Glyma.08G083300 (LRR receptor-like serine/threonine-protein kinase FLS2) was downregulated following infection with *P. pachyrhizi*. Following the perception of flagellin by FLS2, the initial signaling event is likely due to the recruitment of BAK1 into the flagellin receptor complex [41]. Our investigation revealed that the expression of a BAK1 gene (Glyma.17G178700) was specific to susceptible materials, suggesting its potential involvement in the recognition and regulation of pathogen invasion. In *Arabidopsis thaliana*, the MEKK1-MKK1/MKK2-MPK4 mitogen-activated protein (MAP) kinase cascade has been shown to suppress cell death and immune responses [42]. Notably, we observed upregulation of one MEKK1 gene in the resistant materials following 6 h of infection with *P. pachyrhizi*. The defense mechanisms of plants against pathogen attacks necessitate significant transcriptional reprogramming, which is under the regulation of WRKY transcription factors. Previous research has established the involvement of OsWRKY80, a positive regulator, in rice’s defense response against the sheath blight pathogen *R. solani* [43]. Our findings indicate that six WRKY transcription factors were upregulated in resistant material at 6 h post-infection with *P. pachyrhizi*, followed by a downregulation at 10 days post−infection. Additionally, seven WRKY transcription factors were exclusively upregulated in resistant material at 6 h post−infection. At 6 h post−inoculation, only two WRKY transcription factors exhibited upregulation exclusively in susceptible material. These transcription factors are believed to have significant involvement in the process of PTI and effector−triggered immunity during the interaction between soybean and *P. pachyrhizi*. These discoveries offer novel insights into the potential molecular mechanisms underlying soybean resistance to Asian soybean rust.

### 3.3. NLR Genes

ETI is the secondary response to pathogen invasion. Pathogens have the capacity to suppress PTI by injecting effector proteins into plant cells through secretion systems [44]. To counteract the effect of effector proteins, plants have developed specific resistance mechanisms mediated by “R” genes [45]. These genes encode five distinct types of R proteins that recognize effectors from various pathogens, with the largest type encoding the NLR protein. NLRs play a crucial role in the recognition of pathogen effectors and the initiation of immune responses [46]. This present study revealed that 45 NLR genes exhibited differential expression in the plant–pathogen interaction pathway between infected and non−infected resistant and susceptible materials (Supplementary Table S5). Specifically, 28 NLR genes were upregulated in the resistant materials, while 17 NLR genes were upregulated in the susceptible materials. Additionally, 12 NLRs were exclusively expressed in the resistant materials. Upon analysis of the identified resistance locus for *Phakopsora pachyrhizi*, it was observed that the Rpp1 locus region contained a cluster of NLR genes, whose protein sequences exhibited homology with BED finger NLR resistance protein in black cottonwood and putative disease resistance protein RPM1 in castor bean [47]. Additionally, the *Rpp3* region in Hyuuga was found to comprise 7 NLR genes [48]. Furthermore, the NLR resistance genes in the *Rpp4* region displayed a high degree of homology with the downy mildew resistance genes found in lettuce [10]. Therefore, a lot of evidence showed that SBR resistance genes in soybean might be found in the NLR gene family; these differentially expressed NLR genes in SX6907 may be candidates for resistance to Asian soybean rust.

### 3.4. Flavonoid Biosynthesis and Disease Resistance

The flavonoid, a secondary metabolite, plays a crucial role in various plant functions, particularly in its ability to combat pathogens, which has garnered increasing attention [49]. In tomato roots, flavonoids contribute to the systemic defense response against the gray mold induced by oligogalacturonide, while the isoflavones are essential in R gene−mediated resistance to soybean blight [50]. The downregulation of the isoflavone synthetase gene via RNA interference in soybean roots results in a significant reduction of isoflavone accumulation by 95%, rendering the plant more susceptible to pathogens [51]. In this investigation, the differential expression of 38 genes involved in the flavonoid biosynthesis pathway was examined in response to *P. pachyrhizi* infection in both resistant and susceptible materials (Supplementary Table S6). Notably, the upregulation of one specific dihydroflavonol 4−reductase gene (Glyma.17G173200) was observed in susceptible materials. Dihydroflavonol 4−reductase (DFR) is a crucial enzyme in the flavonoid pathway, responsible for the synthesis of anthocyanins, catechins, and procyanidins [52]. Previous research has demonstrated that the overexpression of the DFR gene can enhance anthracnose resistance in tobacco and promote the accumulation of flavonoids in resistant leaves [53]. This finding diverges somewhat from our own results, prompting further investigation into the relationship between DFR and plant disease resistance. Isoflavone reductase (IFR) is an enzyme that plays a role in the biosynthesis of isoflavonoids in plants. Previous research has demonstrated that the constitutive expression of GmIFR enhances resistance against *P. sojae* [54], while the induction of a rice isoflavone reductase-like gene, OsIRL, is triggered by rice blast fungal elicitor [55]. Our study revealed that four isoflavone reductase genes (Glyma.11G070500, Glyma.11G070600, Glyma.06G317000, and Glyma.11G070200) exhibited an upregulated expression in the resistant materials. These genes have been implicated in the resistance to soybean rust. Caffeoyl−CoA 3-O-methyltransferase (CCoAOMT) is a key enzyme involved in the synthesis of lignin in plants [56]. Previous research has demonstrated that correcting multiallelic mutations in a caffeoyl-CoA O-methyltransferase gene led to increased accumulation of resistance metabolites in Russet Burbank potato [57]. Additionally, a ZmCCoAOMT2 gene has been found to confer quantitative resistance to southern leaf blight and gray leaf spot [58]. In our study, we observed the upregulation of three CCoAOMT2 genes in the resistant materials. The functional significance of the flavonoid biosynthesis and disease-resistant genes identified here needs further investigation.

## 4. Materials and Methods

### 4.1. Plant Genotypes, Inoculations, and Experimental Conditions

**Figure 10:**
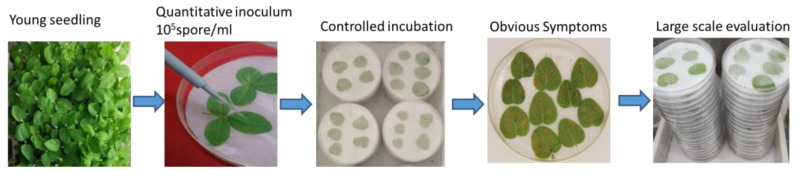
A simple diagram of the inoculation process.

SX6907 is a highly resistant germplasm and has an immune response against *P. pachyrhizi* isolate SS4 [7]. The high-yield variety Tianlong 1, developed by the Oil Crops Research Institute of the Chinese Academy of Agricultural Sciences (OCRI), is susceptible to the *P. pachyrhizi* isolate SS4. Soybean seeds were grown in the greenhouse at 24–26 °C and with a photoperiod of 18/6 h. After 14 days, the fully expanded primary leaves of the seedlings were collected and used for inoculation of the *P. pachyrhizi* isolate SS4. The inoculation method was carried out as described by Chen et al. [21]: The urediniospores were routinely multiplied on Tianlong 1 leaves and collected in tubes. Tween 20 (0.01% *v*/*v*) was used to adjust the urediniospore suspension to 10^5^ urediniospores per ml, followed by placing the detached leaves on a wet filter paper-padded plate. The upper surface of the blade was covered with a layer of filter paper. Five to six leaves were placed on each plate. Each leaf was inoculated with four drops of urediniospore suspension at 5 μL per drop. On the first night after inoculation, the leaves were stored at 24 °C and 70% relative humidity under a 12/12 h photoperiod in a growth chamber. Each day, 1–2 mL of water was added to keep the filter paper moist (Figure 10). Then, the soybean leaves were harvested at four growth stages after infection: 0 h, 6 h, 24 h, and 10 d, respectively. Three biological replicates were collected for each sample.

### 4.2. Observation by Laser Confocal Microscopy

A total of three inoculated soybean leaves were harvested at 6 h, 24 h, and 10 d after inoculation for histopathological analysis. Soybean leaf segments of 2–3 cm^2^ were cut from the center of each inoculated leaf. A solution of ethanol/trichloromethane (3:1, *v*/*v*) containing 0.15% (*w*/*v*) trichloroacetic acid was used to fix and decolorize leaf sections for three to five days. A saturated chloral hydrate solution was used to clear the specimens until the leaf tissues were translucent. For microscopy, cleared leaf segments were stained with 0.1% wheat germ agglutinin (WGA) with Alexa Fluor488 (Thermo Fisher Scientific, Waltham, MA, USA) for 5–10 min, washed with distilled water, and viewed under differential interference contrast optics and UV light. Microscopy examinations were completed with a Zeiss confocal microscope.

### 4.3. RNA Extraction

We extracted total RNA from the tissue with TRIzol^®^ Reagent (Thermo Fisher Scientific, Waltham MA, USA) and removed genomic DNA with DNase I (TaKaRa, Dalian, China). After that, the RNA quality was determined using the 2100 Bioanalyser (Agilent, Technologies, Palo Alto, CA, USA) and quantified using the ND-2000 (NanoDrop Technologies, Wilmington, NE, USA). A high-quality RNA sample (OD260/280 = 1.8~2.2, OD260/230 ≥ 2.0, RIN ≥ 6.5, 28S:18S ≥ 1.0, >10 μg) was used to construct the sequencing library.

### 4.4. Library Preparation and Illumina Hiseq Sequencing

With 1 g of total RNA, the RNA-seq transcriptome libraries were prepared using Illumina’s TruSeqTM RNA sample preparation kit (San Diego, CA, USA). After completing a polyA selection by oligo(dT) beads, the messenger RNA was isolated and fragmented with a fragmentation buffer. Illumina’s protocol was followed for the cDNA synthesis, end repair, A-base addition, and ligation of the Illumina-indexed adapters. The cDNA libraries were then size-selected for 200–300 bp target fragments on the 2% Low Range Ultra Agarose, followed by 15 PCR cycles using the Phusion DNA polymerase (NEB). Paired-end libraries were quantified by TBS380 and then sequenced by the Illumina NovaSeq 6000 (150 bp × 2, Shanghai BIOZERON Co., Ltd., Shanghai, China).

### 4.5. Reads Quality Control and Mapping

Raw paired-end reads were trimmed and quality controlled with Trimmomatic (SLIDINGWINDOW: 4:15 MINLEN:75) (version 0.36, http://www.usadellab.org/cms/uploads/supplementary/Trimmomatic, accessed on 2 March 2020). After that, clean reads were aligned separately to the reference genome using the Hisat2 in orientation mode (https://ccb.jhu.edu/software/hisat2/index.shtml, accessed on 2 March 2020) software. We mapped with default parameters using this software. The quality assessment of these data was taken by qualimap_v2.2.1 (used htseq, https://htseq.readthedocs.io/en/release_0.11.1/, accessed on 2 March 2020) to count each gene read.

### 4.6. Differential Expression Analysis and Functional Enrichment

We calculated the expression levels of each gene using the fragments per kilobase of exon per million mapped reads (FRKM) method to identify the DEGs between the two samples. The R statistical package edgeR (Empirical Analysis of Digital Gene Expression in R, http://www.bioconductor.org/packages/release/bioc/html/edgeR.html/, accessed on 20 March 2020) was used for the differential expression analysis. The DEGs between two samples were selected according to the following criteria: logarithmic fold changes greater than 2 and a false discovery rate (FDR) of less than 0.05. Goatools (https://github.com/tanghaibao/Goatools, accessed on 20 March 2020) and KOBAS (http://kobas.cbi.pku.edu.cn/home.do, accessed on 20 March 2020) were used to analyze the GO functional enrichment and KEGG pathway analysis of the differentially expressed genes. When the Bonferroni-corrected *p*-value for the DEGs was less than 0.05, they were significantly enriched in GO terms and metabolic pathways.

### 4.7. WGCNA Construct

For the WGCNA, Pearson’s correlation coefficient absolute value is used to measure the gene co-expression in the network. This absolute value is then raised to a power to generate the adjacency matrix. Based on the adjacency matrix, the topological overlap distance is then clustered based on the average linkage hierarchical clustering. Our modules were defined using the cutreeDynamic function with a minimum module size of 30 genes. The mergeCloseModules function was used to merge the modules with eigengene distances of less than 0.25. Biologically significant edges were determined for each module by empirically setting the weight value to 0.10. The biological significance of each module was explored through correlation analyses between the module eigengenes and measured agronomic traits. As a rule of thumb, a gene whose edges comprise over 15% of a module is considered the hub gene.

## 5. Conclusions

This study presents the first large-scale comparative transcriptomic profiling of resistant and susceptible soybean genotypes in response to the invasion of *P. pachyrhizi*. The results revealed a complex and massive gene network response, providing insight into the mechanisms directing resistance to *P. pachyrhizi* in soybeans. The analysis suggests that the NB-ARC domain-containing disease-resistant genes and cysteine-rich receptor-like protein kinase genes play an essential role in understanding pathogen invasion through a downstream resistance mechanism. *P. pachyrhizi* infection was associated with diverse plant defense response TF families, such as ERFs, WRKY, MYB, and bHLH. Furthermore, the genes involved in the Ca^2+^ signaling pathway, MAPK signaling pathway, and flavonoid biosynthesis were upregulated in the resistant materials, thereby inducing systemic resistance. Moreover, the co-expression network analysis (WGCNA) identified that two modules were mainly enriched in RNA processing, plant-type hypersensitive responses, negative regulation of cell growth, and programmed cell death. In summary, this study provided an important resource to mine disease-resistant genes and will presumably provide a new theoretical basis for the optimization and innovation of genetic breeding for soybean crops.

## Figures and Tables

**Table 1:** Summary statistics of RNA-seq data and mapping results.

Group	Sample Name	Clean Reads (M)	Clean Bases (G)	Mapped Reads (%)	Uniquely Mapped (%)	No. of Mapped Genes
1	R-0 hpi (Rep 1)	39.0659	5.8599	93.15	89.68	39,658
	R-0 hpi (Rep 2)	40.9507	6.1426	92.38	90.30	39,736
	R-0 hpi (Rep 3)	39.2121	5.8818	92.11	89.14	38,620
2	R-6 hpi (Rep 1)	40.6590	5.9497	90.14	87.11	37,646
	R-6 hpi (Rep 2)	37.8276	5.6741	70.75	69.25	37,054
	R-6 hpi (Rep 3)	39.6647	6.0988	91.19	88.99	37,396
3	R-24 hpi (Rep 1)	38.8942	5.7379	92.74	90.35	38,848
	R-24 hpi (Rep 2)	40.3832	6.0575	92.91	90.82	38,635
	R-24 hpi (Rep 3)	38.2529	5.8341	78.34	76.65	38,307
4	R-10 dpi (Rep 1)	41.4718	5.7205	80.99	79.11	38,309
	R-10 dpi (Rep 2)	40.0860	6.0129	82.90	80.90	37,731
	R-10 dpi (Rep 3)	40.0860	6.2208	92.61	90.39	38,463
5	S-0 hpi (Rep 1)	45.1133	6.7670	91.74	88.53	37,033
	S-0 hpi (Rep 2)	43.1935	6.4790	91.68	89.38	38,407
	S-0 hpi (Rep 3)	41.1932	6.1790	91.16	87.72	35,172
6	S-6 hpi (Rep 1)	42.2855	6.2377	91.19	88.56	37,847
	S-6 hpi (Rep 2)	43.3958	6.5094	91.02	88.53	40,092
	S-6 hpi (Rep 3)	41.5849	6.3428	90.30	88.08	38,527
7	S-24 hpi (Rep 1)	47.2346	6.6490	92.85	90.16	39,249
	S-24 hpi (Rep 2)	37.8453	5.6768	92.56	89.60	31,962
	S-24 hpi (Rep 3)	44.3266	7.0852	91.56	89.30	38,696
8	S-10 dpi (Rep 1)	38.9951	6.4775	86.10	83.94	37,657
	S-10 dpi (Rep 2)	39.0004	5.8501	73.21	56.29	35,421
	S-10 dpi (Rep 3)	43.1835	5.8493	80.69	78.55	40,234

**Table 2:** Summary statistics of important differentially expressed genes at all 6 hpi, 24 hpi, and 10 dpi.

Gene ID	Description	Point-in-Time	
Glyma.18G087400, Glyma.03G087800, Glyma.03G047000, Glyma.03G054100, Glyma.14G205000, Glyma.18G082800, Glyma.14G199400, Glyma.18G088500, Glyma.07G063600, Glyma.09G208900, Glyma.07G063700, Glyma.01G046900, Glyma.06G259400, Glyma.16G214800, Glyma.16G215000	LRR and NB-ARC domain-containing disease-resistant proteins	6 hpi; 24 hpi; 10 dpi	upregulated
Glyma.17G214600; Glyma.17G214700	Rust resistance kinase Lr10 isoform	6 hpi; 24 hpi; 10 dpi	upregulated
Glyma.16G214000, Glyma.16G210600, Glyma.03G047700, Glyma.13G190000, Glyma.16G214500, Glyma.13G187900, Glyma.15G233200, Glyma.18G280300, Glyma.06G259800, Glyma.18G281500, Glyma.18G086400, Glyma.13G190400, Glyma.16G085400, Glyma.18G086900, Glyma.18G086600, Glyma.03G088000, Glyma.03G088100	LRR and NB-ARC domain-containing disease-resistant proteins	6 hpi; 24 hpi; 10 dpi	downregulated
Glyma.12G233000, Glyma.18G198800, Glyma.18G268000, Glyma.16G185100, Glyma.09G107600, Glyma.13G188800, Glyma.06G226700	LRR receptor protein kinase	6 hpi; 24 hpi; 10 dpi	downregulated
Glyma.13G033500, Glyma.13G033400	Rust resistance kinase lr10-related protein	6 hpi; 24 hpi; 10 dpi	downregulated
Glyma.05G127400, Glyma.04G220200	Programmed cell death protein	6 hpi; 24 hpi; 10 dpi	downregulated
Glyma.16G211500, Glyma.15G187300	TMV resistance protein N isoform	6 hpi; 24 hpi; 10 dpi	downregulated

**Table 3:** Gene ontology enrichment of DEGs (biological process) in SX6907 compared with tianlong1 under *P. pachyrhizi* infection at 0 hpi, 6 hpi, 24 hpi, and 10 dpi. Number of DEGs contained in respective categories is given as a *p*-value. Only categories top 10 and *p* < 0.05 are displayed.

GO Term (Biological Process)	Cluster Frequency	*p*-Value
0 hpi		
defense response	58 out of 392 genes, 14.8%	2.96 × 10^−21^
response to stress	72 out of 392 genes, 18.4%	2.18 ×10^−7^
response to stimulus	100 out of 392 genes, 25.5%	1.2 ×10^−3^
6 hpi		
defense response	106 out of 1202 genes, 8.8%	8.55 × 10^−21^
photosynthesis, light harvesting in photosystem I	17 out of 1202 genes, 1.4%	3.97 × 10^−12^
photosynthesis, light harvesting	19 out of 1202 genes, 1.6%	3.80 × 10^−10^
protein-chromophore linkage	19 out of 1202 genes, 1.6%	6.04 × 10^−9^
secondary metabolic process	55 out of 1202 genes, 4.6%	1.85 × 10^−7^
response to stress	163 out of 1202 genes, 13.6%	1.41 × 10^−6^
photosynthesis, light reaction	20 out of 1202 genes, 1.7%	7.40 × 10^−6^
response to stimulus	270 out of 1202 genes, 22.5%	1.05 × 10^−5^
flavonoid metabolic process	34 out of 1202 genes, 2.8%	4.57 × 10^−5^
flavonoid biosynthetic process	33 out of 1202 genes, 2.7%	1.12 × 10^−3^
24 hpi		
defense response	47 out of 320 genes, 14.7%	7.71 × 10^−17^
response to stress	66 out of 320 genes, 20.6%	6.07 × 10^−9^
response to stimulus	86 out of 320 genes, 26.9%	5.62 × 10^−3^
10 dpi		
defense response	172 out of 2179 genes, 7.9%	7.67 × 10^−30^
secondary metabolic process	108 out of 2179 genes, 5.0%	1.40 × 10^−19^
multi-organism process	66 out of 2179 genes, 3.0%	2.21 × 10^−12^
protein phosphorylation	307 out of 2179 genes, 14.1%	4.79 × 10^−12^
secondary metabolite biosynthetic process	75 out of 2179 genes, 3.4%	5.15 × 10^−12^
oxidation-reduction process	374 out of 2179 genes, 17.2%	4.85 × 10^−11^
cell recognition	42 out of 2179 genes, 1.9%	3.47 × 10^−10^
pollen–pistil interaction	42 out of 2179 genes, 1.9%	3.47 × 10^−10^
recognition of pollen	42 out of 2179 genes, 1.9%	3.47 × 10^−10^
response to biotic stimulus	42 out of 2179 genes, 1.9%	1.10 × 10^−9^

**Table 4:** DEGs enriched in the pathways related to plant defense at 6 hpi, 24 hpi, and 48 dpi.

Pathways	6 hpi	24 hpi	10 dpi
Plant–pathogen interaction	115	45	78
Plant hormone signal transduction	53	10	21
Flavonoid biosynthesis	29	5	14
Phenylpropanoid biosynthesis	47	11	21
Galactose metabolism	11	4	8
Photosynthesis-antenna proteins	19	0	2
Ubiquitin-mediated proteolysis	15	3	7
ABC transporters	23	2	7
Brassinosteroid biosynthesis	1	1	2
Proteasome	2	0	2
Phenylalanine metabolism	9	1	21
Protein processing in endoplasmic reticulum	48	12	17
Flavone and flavonol biosynthesis	7	1	5

## Data Availability

The data disclosed in this study can be accessed through the article or Supplementary Materials provided here.

## Supplementary Materials

The supporting information can be downloaded at: https://www.mdpi.com/article/10.3390/ijms241713450/s1.
