# Supplementary material for: RNA-Seq and Comparative Transcriptomic Analyses of Asian Soybean Rust Resistant and Susceptible Soybean Genotypes Provide Insights into Identifying Disease Resistance Genes
Source: Int J Mol Sci. 2023 Aug 30;24(17):13450. doi: 10.3390/ijms241713450 (PMC10487414; doi:10.3390/ijms241713450)
Supplement: Supplementary file 1 [file ijms-24-13450-s001.zip › Supplementary Figure S2.pptx]

## Slide 1
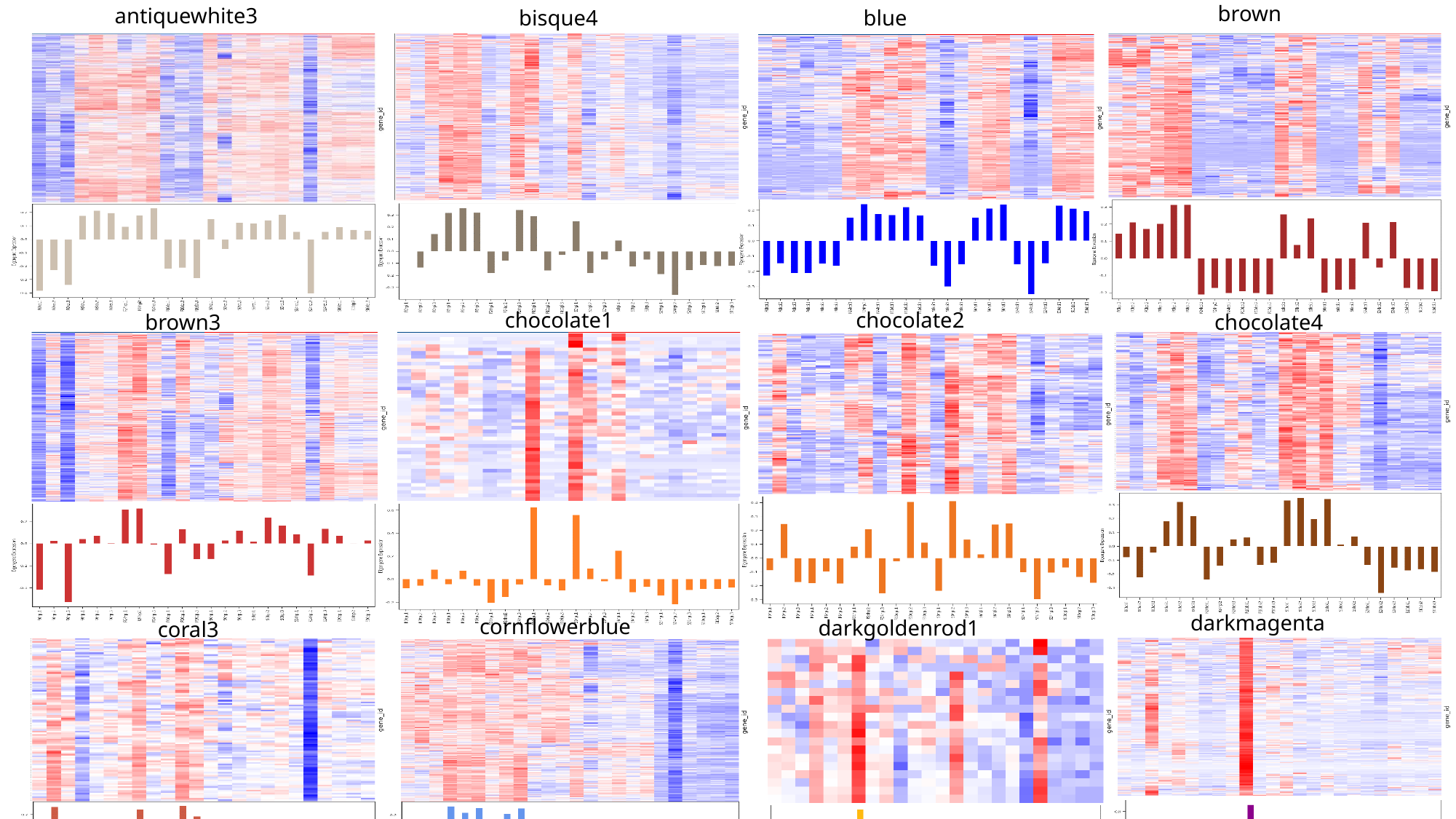

brown
antiquewhite3
bisque4
blue
chocolate1
chocolate2
brown3
chocolate4
darkmagenta
cornflowerblue
darkgoldenrod1
coral3

## Slide 2
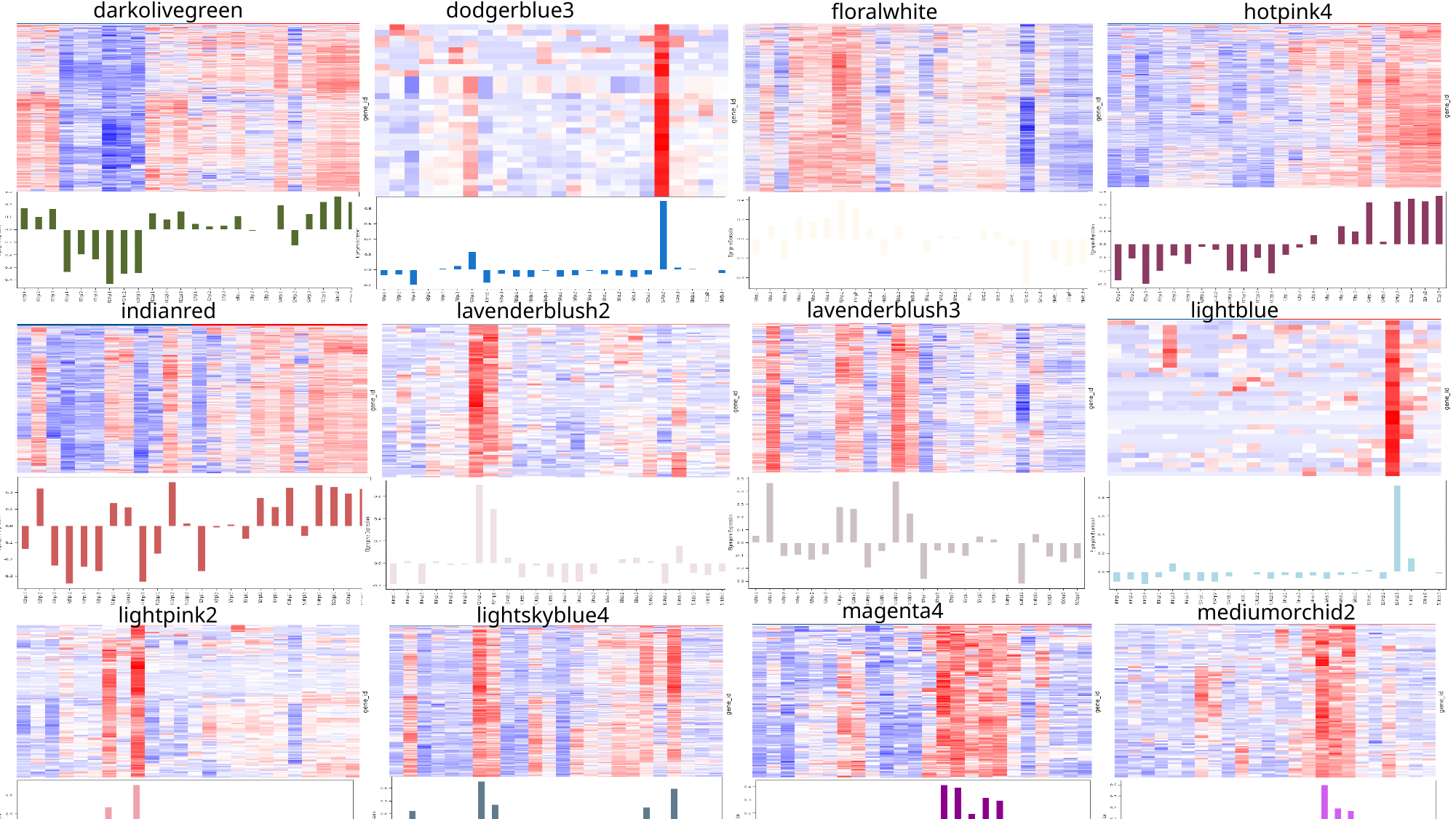

darkolivegreen
dodgerblue3
floralwhite
hotpink4
lavenderblush3
lightblue
indianred
lavenderblush2
magenta4
mediumorchid2
lightskyblue4
lightpink2

## Slide 3
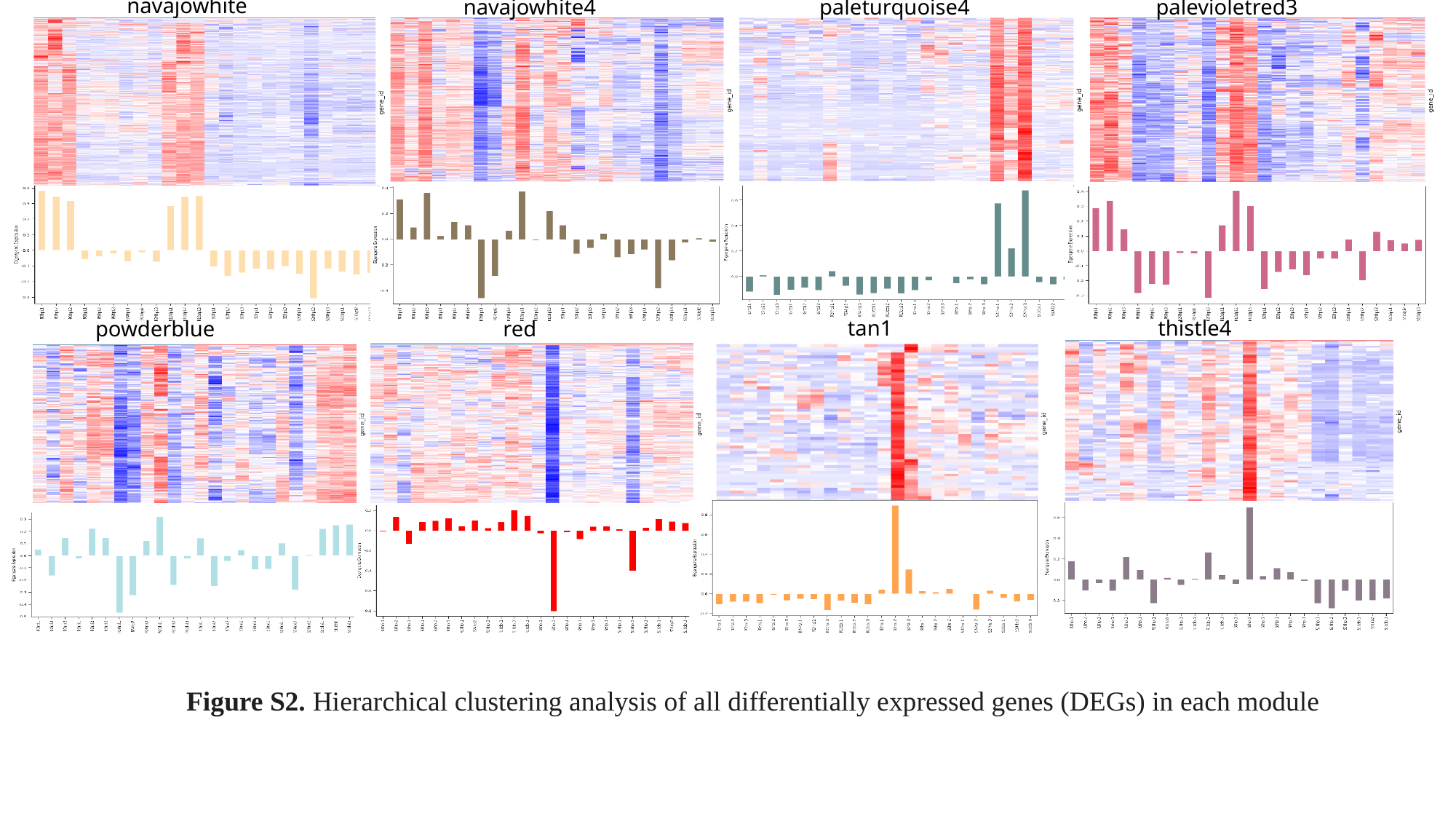

navajowhite
navajowhite4
palevioletred3
paleturquoise4
tan1
thistle4
powderblue
red
Figure S2. Hierarchical clustering analysis of all differentially expressed genes (DEGs) in each module
