# Supplementary figures and images for: RNA-Seq and Comparative Transcriptomic Analyses of Asian Soybean Rust Resistant and Susceptible Soybean Genotypes Provide Insights into Identifying Disease Resistance Genes
Source: Int J Mol Sci. 2023 Aug 30;24(17):13450. doi: 10.3390/ijms241713450 (PMC10487414; doi:10.3390/ijms241713450)

## Slide 1
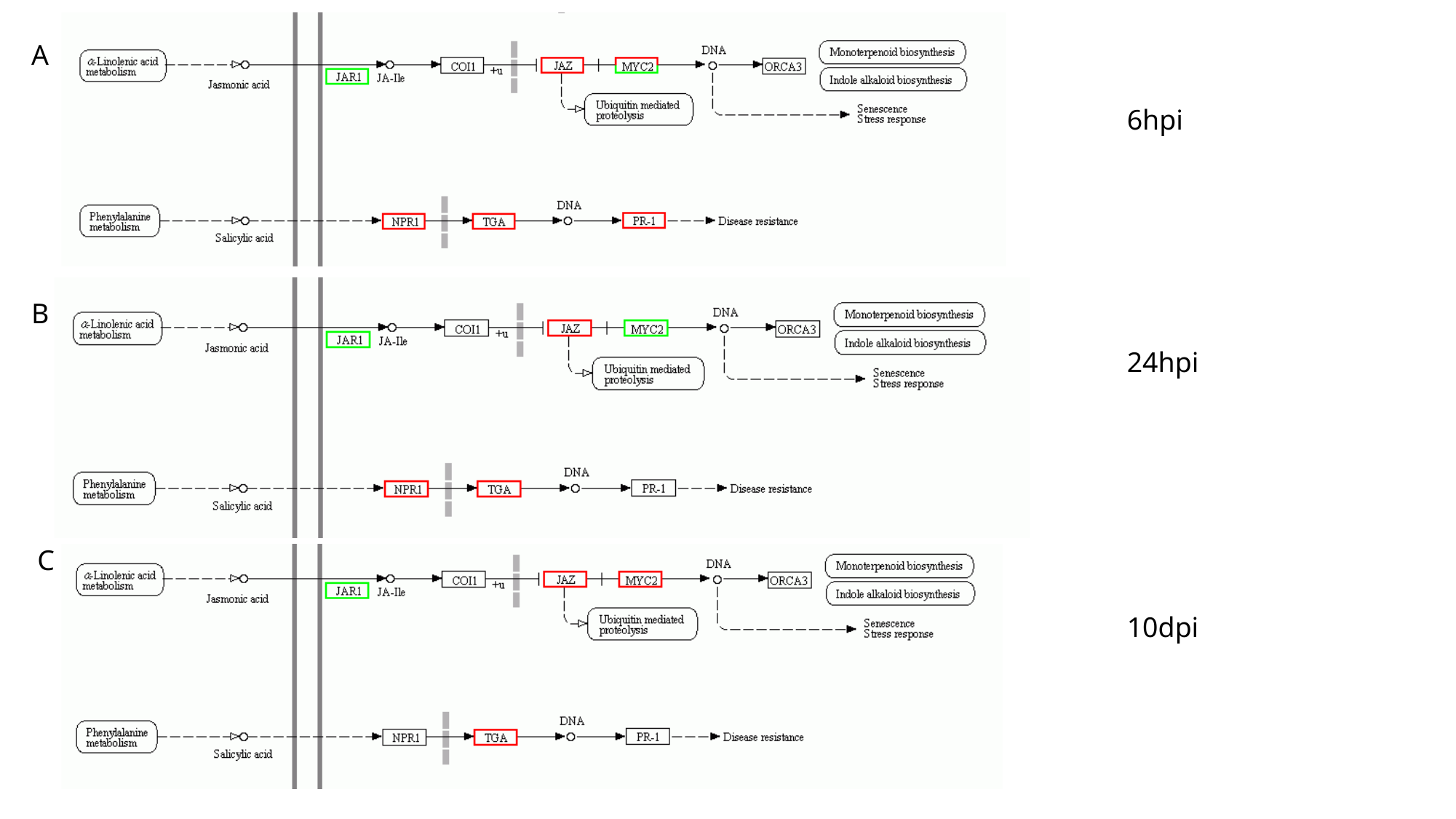

A
6hpi
B
24hpi
C
10dpi

## Slide 2
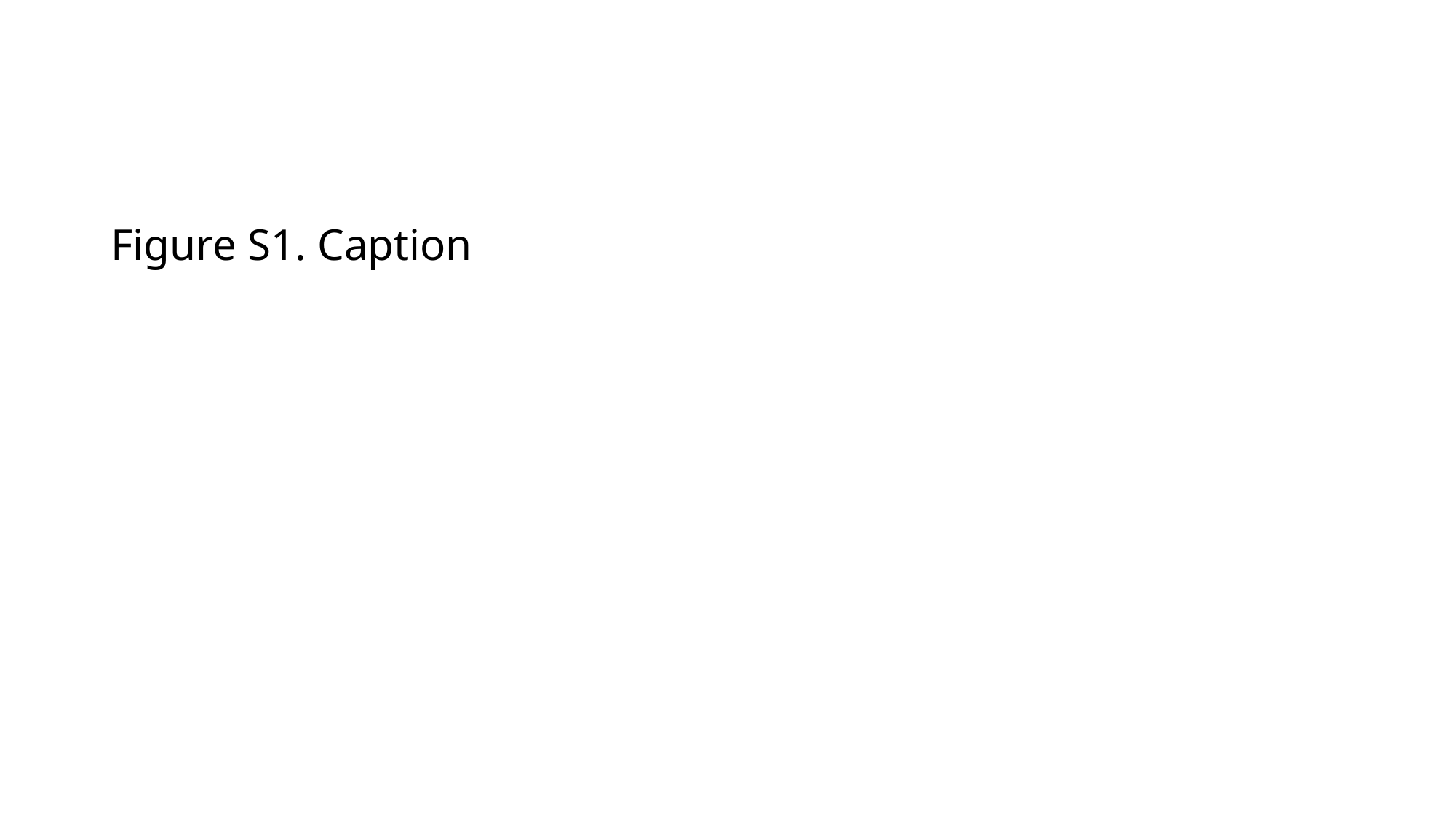

Figure S1. Caption

Supplement: Supplementary file 1 [file ijms-24-13450-s001.zip › Supplementary Figure S1.pptx]

## Slide 1
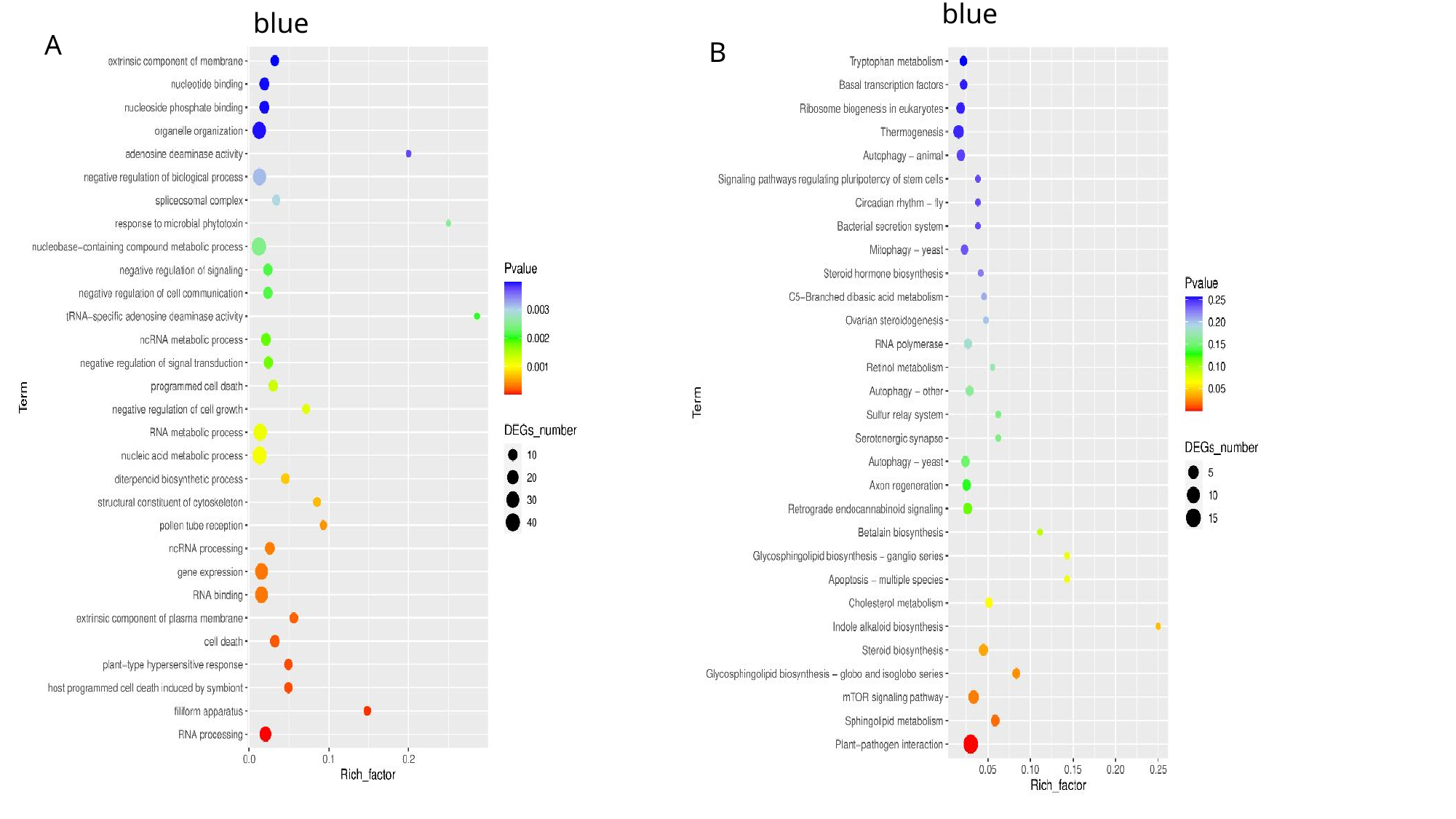

blue
blue
A
B

## Slide 2
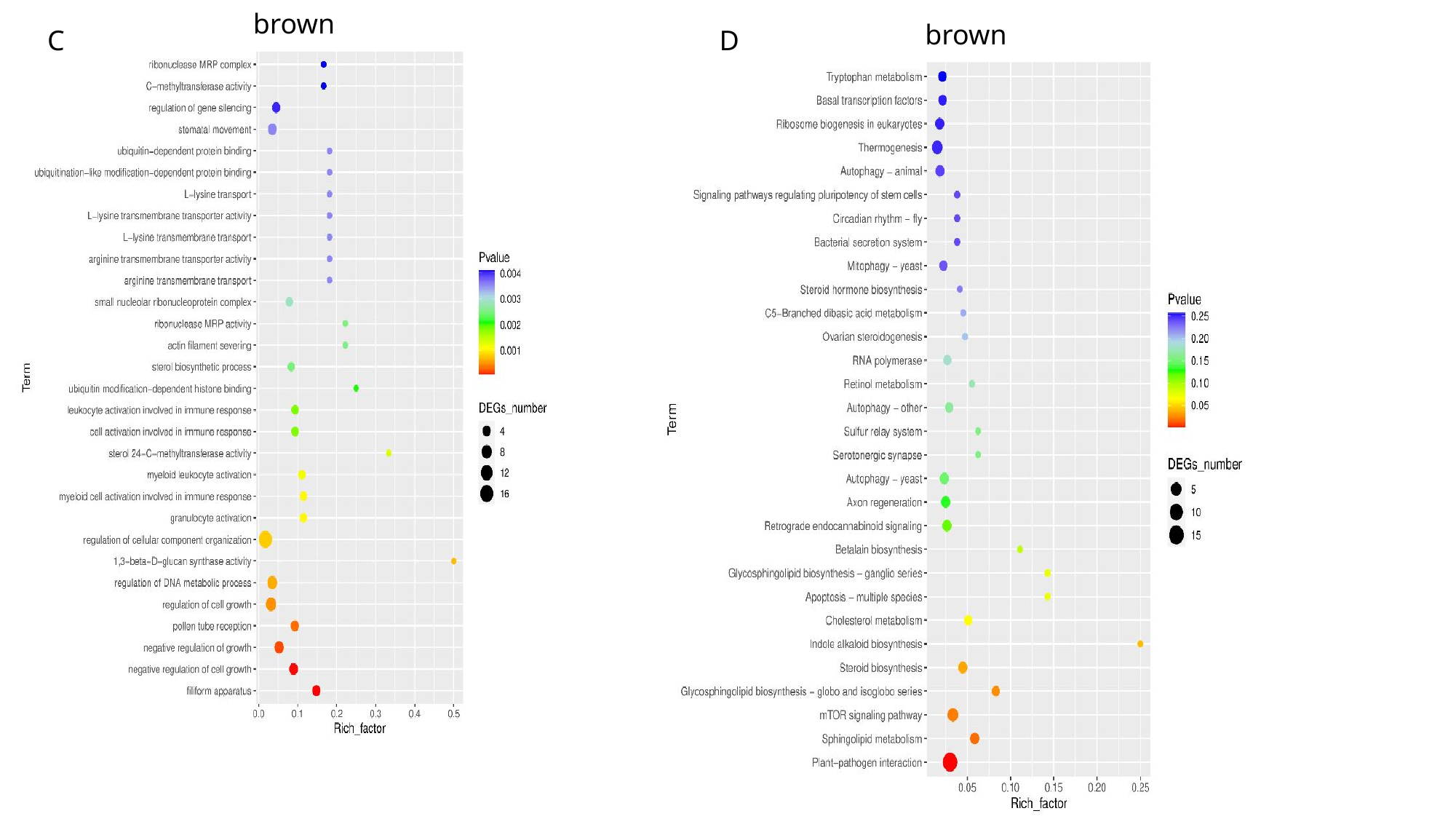

brown
brown
C
D

## Slide 3
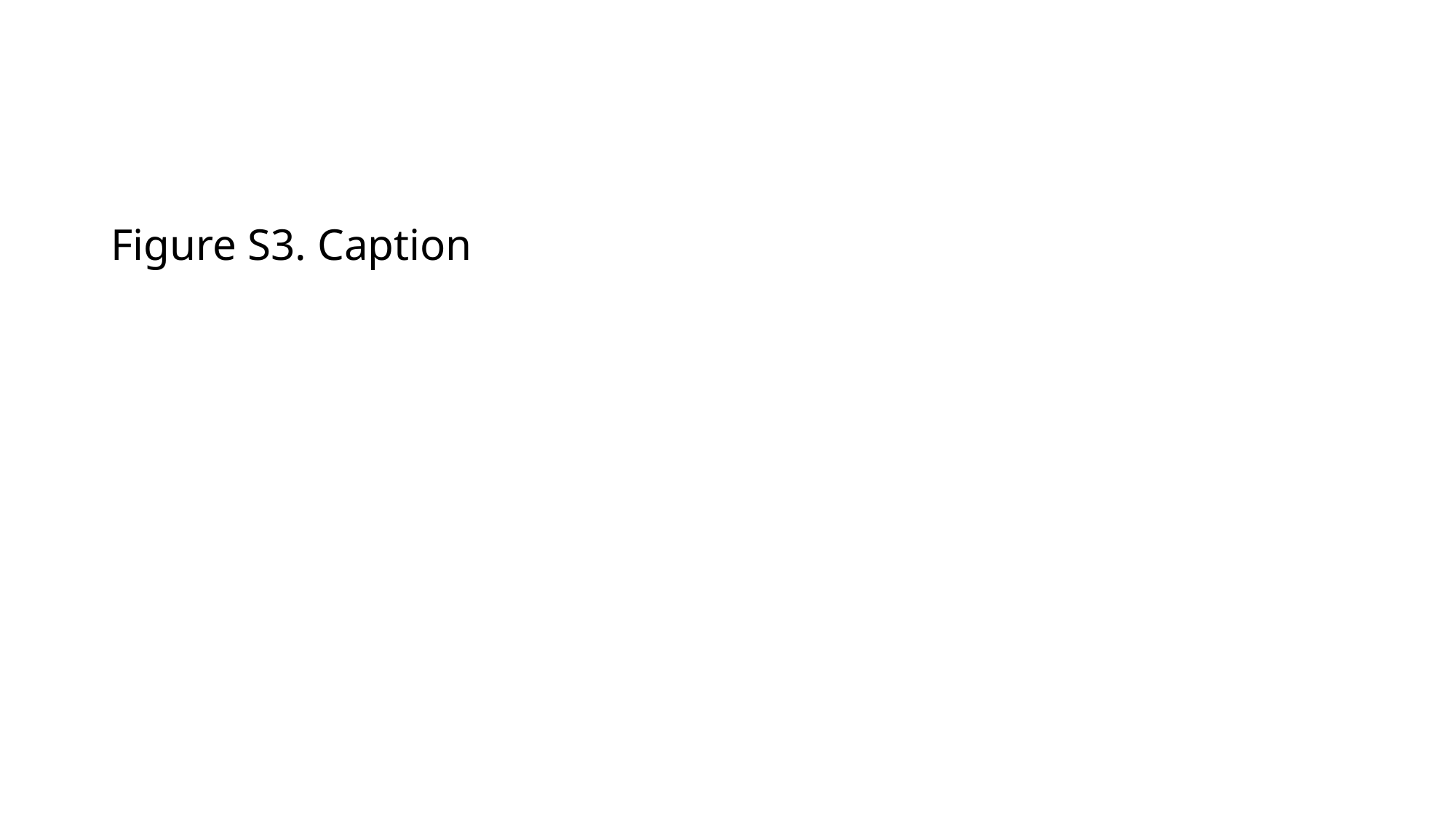

Figure S3. Caption

Supplement: Supplementary file 1 [file ijms-24-13450-s001.zip › Supplementary Figure S3.pptx]

## Slide 1
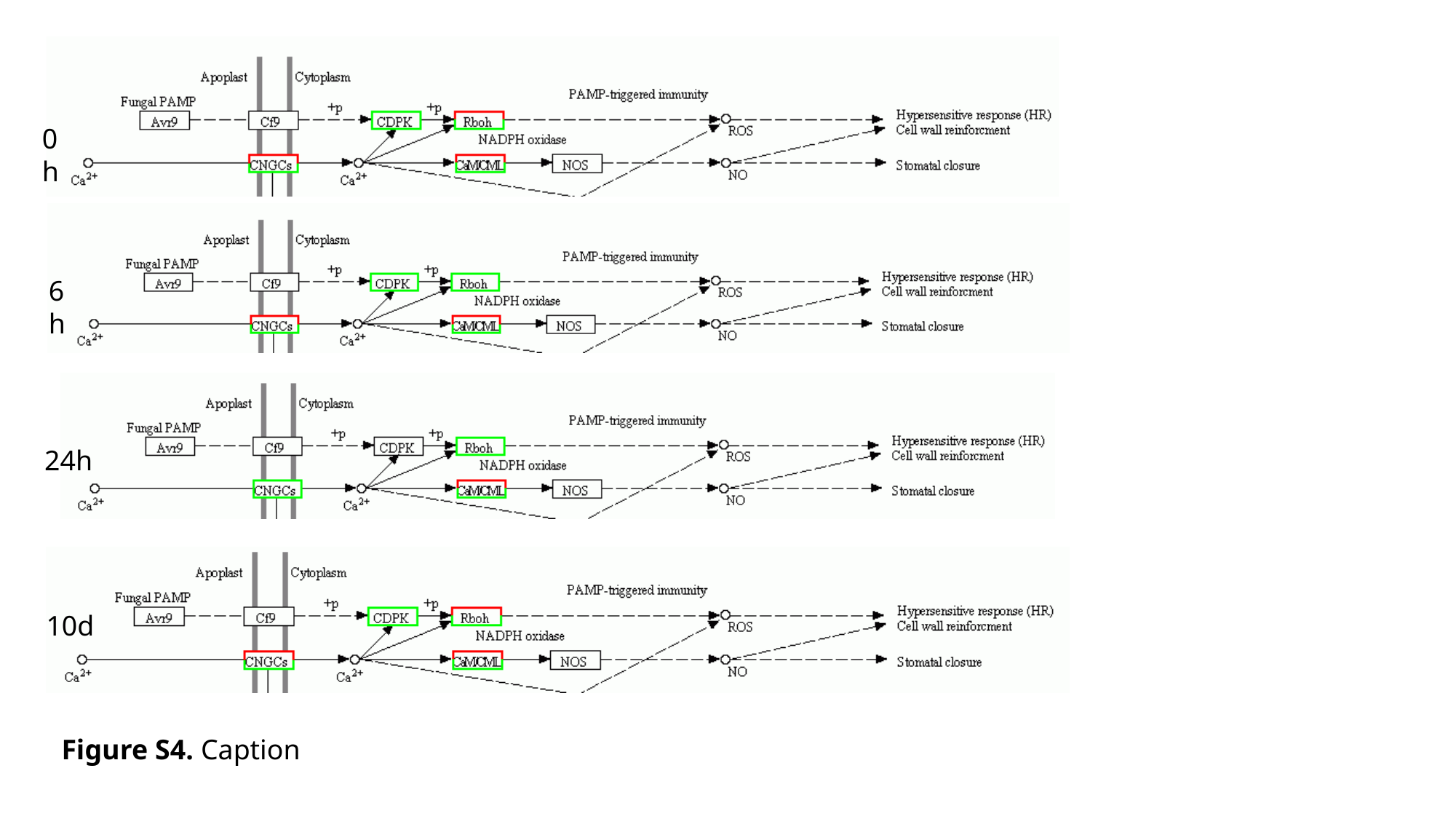

0h
6h
24h
10d
Figure S4. Caption

Supplement: Supplementary file 1 [file ijms-24-13450-s001.zip › Supplementary Figure S4.pptx]
